# Supplementary material for: Empirical methods for controlling false positives and estimating confidence in ChIP-Seq peaks
Source: BMC Bioinformatics. 2008 Dec 5;9:523. doi: 10.1186/1471-2105-9-523 (PMC2628906; doi:10.1186/1471-2105-9-523)
Supplement: Additional File 1 — A variety of html documents from the USeq web site detailing the available applications, their best usage, output file type descriptions, command line menus, etc. [file 1471-2105-9-523-S1.zip › USeqUserGuides/JavaDoc.zip!/JavaDoc/bioroot/strain/StrainModify.html]

StrainModify


|  |  |  |
| --- | --- | --- |
| **Overview** \| **Package** \| **Class** \| **Use** \| **Tree** \| **Deprecated** \| **Index** \| **Help** | |  |
| **PREV CLASS**   **NEXT CLASS** | **FRAMES**    **NO FRAMES**     **All Classes** |  |
| SUMMARY: NESTED \| FIELD \| CONSTR \| METHOD | DETAIL: FIELD \| CONSTR \| METHOD |  |


---


## bioroot.strain Class StrainModify

```
java.lang.Object
  javax.servlet.GenericServlet
      javax.servlet.http.HttpServlet
          bioroot.strain.StrainModify
```

**All Implemented Interfaces:**: java.io.Serializable, javax.servlet.Servlet, javax.servlet.ServletConfig

---

``` public class StrainModify extends javax.servlet.http.HttpServlet ```

Servlet to modify strains using StrainBeans referenced from a Session object.

**Author:**
:   nix

**See Also:**: Serialized Form

---

**Constructor Summary**

`StrainModify()`


| **Method Summary** | |
| --- | --- |
| `static java.lang.String` | `deleteStrain(StrainBean strainBean, UserBean userBean, DBUtil bioRoot)`             Attempts to delete an strain, if sucessful, returns a "" String, otherwise an error message. |
| `void` | `doGet(javax.servlet.http.HttpServletRequest request, javax.servlet.http.HttpServletResponse response)` |
| `void` | `doPost(javax.servlet.http.HttpServletRequest request, javax.servlet.http.HttpServletResponse response)` |
| `static boolean` | `subtractAndSetStrainBeans(StrainBean[] strainBeans, javax.servlet.http.HttpSession session)`             Subtracts a bean from the StrainBean[] and resets it in strainBeansToEdit |
| `static java.lang.String` | `updateStrain(StrainBean bean, UserBean userBean, java.util.HashMap nameValue, javax.servlet.http.HttpServletRequest request, DBUtil bioRoot)`             Attempts to update a StrainBean in the database, only checks that the name is unique if it has changed. |
| `static boolean` | `updateStrainCommonAccessFields(StrainBean bean, UserBean userBean, java.util.HashMap nameValue, DBUtil bioRoot)`             Just update the commonly accessible fields: restock, lastUser, remainingAliquots, comments |

**Methods inherited from class javax.servlet.http.HttpServlet**

`service`

**Methods inherited from class javax.servlet.GenericServlet**

`destroy, getInitParameter, getInitParameterNames, getServletConfig, getServletContext, getServletInfo, getServletName, init, init, log, log`

**Methods inherited from class java.lang.Object**

`equals, getClass, hashCode, notify, notifyAll, toString, wait, wait, wait`

**Constructor Detail**

### StrainModify

```
public StrainModify()
```


**Method Detail**

### doGet

```
public void doGet(javax.servlet.http.HttpServletRequest request,
                  javax.servlet.http.HttpServletResponse response)
           throws javax.servlet.ServletException,
                  java.io.IOException
```

:   **Overrides:**: `doGet` in class `javax.servlet.http.HttpServlet`
:   **Throws:**: `javax.servlet.ServletException`: `java.io.IOException`

---


### doPost

```
public void doPost(javax.servlet.http.HttpServletRequest request,
                   javax.servlet.http.HttpServletResponse response)
            throws javax.servlet.ServletException,
                   java.io.IOException
```

:   **Overrides:**: `doPost` in class `javax.servlet.http.HttpServlet`
:   **Throws:**: `javax.servlet.ServletException`: `java.io.IOException`

---


### deleteStrain

```
public static java.lang.String deleteStrain(StrainBean strainBean,
                                            UserBean userBean,
                                            DBUtil bioRoot)
```

:   Attempts to delete an strain, if sucessful, returns a "" String, otherwise an error message.

---


### updateStrainCommonAccessFields

```
public static boolean updateStrainCommonAccessFields(StrainBean bean,
                                                     UserBean userBean,
                                                     java.util.HashMap nameValue,
                                                     DBUtil bioRoot)
```

:   Just update the commonly accessible fields: restock, lastUser, remainingAliquots, comments

---


### updateStrain

```
public static java.lang.String updateStrain(StrainBean bean,
                                            UserBean userBean,
                                            java.util.HashMap nameValue,
                                            javax.servlet.http.HttpServletRequest request,
                                            DBUtil bioRoot)
```

:   Attempts to update a StrainBean in the database, only checks that the name is unique if it has changed.
    If the update is sucessful, the message will be "", otherwise an error message will be returned.

---


### subtractAndSetStrainBeans

```
public static boolean subtractAndSetStrainBeans(StrainBean[] strainBeans,
                                                javax.servlet.http.HttpSession session)
```

:   Subtracts a bean from the StrainBean[] and resets it in strainBeansToEdit


---


|  |  |  |
| --- | --- | --- |
| **Overview** \| **Package** \| **Class** \| **Use** \| **Tree** \| **Deprecated** \| **Index** \| **Help** | |  |
| **PREV CLASS**   **NEXT CLASS** | **FRAMES**    **NO FRAMES**     **All Classes** |  |
| SUMMARY: NESTED \| FIELD \| CONSTR \| METHOD | DETAIL: FIELD \| CONSTR \| METHOD |  |


---
